# Unraveling the rat blood genome-wide transcriptome after oral administration of lavender oil by a two-color dye-swap DNA microarray approach

**DOI:** 10.1016/j.gdata.2016.05.005

**Published:** 2016-05-12

**Authors:** Motohide Hori, Hiroko Kubo, Junko Shibato, Tomomi Saito, Tetsuo Ogawa, Minoru Wakamori, Yoshinori Masuo, Seiji Shioda, Randeep Rakwal

**Affiliations:** a Department of Oral Biology, Graduate School of Dentistry, Tohoku University, Sendai 980-8575, Japan; b Department of Anatomy I, Showa University School of Medicine, 1-5-8 Hatanodai, Shinagawa, Tokyo 142-8555, Japan; c Oriental Aromatherapy College, 5-22-9 Kameari, Katsushika, Tokyo 125-0061, Japan; d Global Research Center for Innovative Life Science, Peptide Drug Innovation, School of Pharmacy and Pharmaceutical Sciences, Hoshi University, 4-41 Ebara 2-chome, Shinagawa, Tokyo 142-8501, Japan; e Department of Physiology, Saitama Medical University, 38 Morohongo Moroyama-machi, Iruma-gun, Saitama 350-0495, Japan; f Laboratory of Neuroscience, Department of Biology, Faculty of Science, Toho University, Funabashi, Chiba 274-8510, Japan; g Faculty of Health and Sport Sciences and Tsukuba International Academy for Sport Studies (TIAS), University of Tsukuba, 1-1-1 Tennodai, Tsukuba 305-8574, Ibaraki, Japan

**Keywords:** Lavender oil ingestion, Rat, Whole blood, Total RNA extraction, Gene expression

## Abstract

Lavender oil (LO) is a commonly used essential oil in aromatherapy as non-traditional medicine. With an aim to demonstrate LO effects on the body, we have recently established an animal model investigating the influence of orally administered LO in rat tissues, genome-wide. In this brief, we investigate the effect of LO ingestion in the blood of rat. Rats were administered LO at usual therapeutic dose (5 mg/kg) in humans, and following collection of the venous blood from the heart and extraction of total RNA, the differentially expressed genes were screened using a 4 × 44-K whole-genome rat chip (Agilent microarray platform; Agilent Technologies, Palo Alto, CA, USA) in conjunction with a two-color dye-swap approach. A total of 834 differentially expressed genes in the blood were identified: 362 up-regulated and 472 down-regulated. These genes were functionally categorized using bioinformatics tools. The gene expression inventory of rat blood transcriptome under LO, a first report, has been deposited into the Gene Expression Omnibus (GEO): GSE67499. The data will be a valuable resource in examining the effects of natural products, and which could also serve as a human model for further functional analysis and investigation.

**Table:** 

Specifications	
Organism/cell line/tissue	*Rattus norvegicus*, Fischer, F334, whole blood
Sex	Male
Sequencer or array type	Agilent whole rat genome microarray G4131F
Data format	Raw data: TXT file, Normalized data: TXT
Experimental factors	Lavender oil (5 mg/kg) group vs. control group (10% ethanol, diluent)
Experimental features	Transcript profiling of differentially expressed genes in rat whole blood following oral ingestion of lavender oil
Consent	Data are publically available
Sample source location	Tokyo, Japan

## 1 Direct link to deposited data

https://www.ncbi.nlm.nih.gov/geo/query/acc.cgi?acc=GSE67499

## 2 Experimental design, materials and methods

### 2.1 Lavender oil (LO) and experimental design for whole blood transcriptome in rat

Our group has been studying the effects of lavender oil (hereafter, LO) on rat model genome-wide [1] using the high-throughput DNA microarray analysis approach [2]. The first transcriptomics study describes the effects of oral ingestion of LO on the expression of genes in the small intestine, spleen and liver [1]. DNA microarray-based profiling using the Agilent platform has been efficiently utilized by our collaborative group to provide a confident inventory of the time-dependent mRNA level changes in global gene expression in diverse tissues in animal models [3], [4], [5], [6], [7]. Among these tissues, a complete study on the whole blood transcriptome analysis was lacking. Experimenting on the blood transcriptome in both animal (rat and mouse) and human subjects for stress and neurodevelopmental disorders (Rakwal and co-workers, unpublished data), we have optimized a protocol for extracting good quality total RNA from whole blood, deep frozen immediately in liquid nitrogen, for downstream DNA microarray analysis, and which is one subject of this present research. Moreover, the genomic data from blood (cells) is becoming an important tool in our search for potential biomarkers for health/disease [8], [9]. Two pitfalls remain, first, a protocol for total RNA extraction from whole deep-frozen blood, and second, demonstration of the transcript profiles in a DNA microarray-based study in a (animal) rat model.

In the present study, utilizing the rat model for an aromatherapy study of essential oil of lavender (LO) as an example, we have investigated the whole blood for genome-wide expression profiling of genes affected by oral intake of LO. Briefly, to explain about LO, the oil distilled from the Labiatae family member — *Lavandula angustifolia* is the source for the commonly used oil in aromatherapy [10], [11]. The essential oil of lavender has been traditionally used in some European countries [1], [12] rather than in Japan or other Asian countries, where aromatherapy is widely used mostly through inhalation of volatile components or application of diluted oil to the skin [1], [13]. Although, and as mentioned in the previous LO paper, in France, Belgium and Germany, LO is used as a form of herbal or natural medicine [1]. To note, a German pharmaceutical company also produces an encapsulated form of LO, “Silexan” and which is sold commercially under the trade name LASEA (W. Spitzner Arzmeimittelfabrik GmbH, Ettlingen, Germany). Although the safety aspects of ingesting essential oils might still be of concern, it is to note that a clinical study has shown positive beneficial effects of Silexan [14]. This was also reason for us to carry out the LO study using rats who ingested the essential oil [1].

**Fig. 1:**
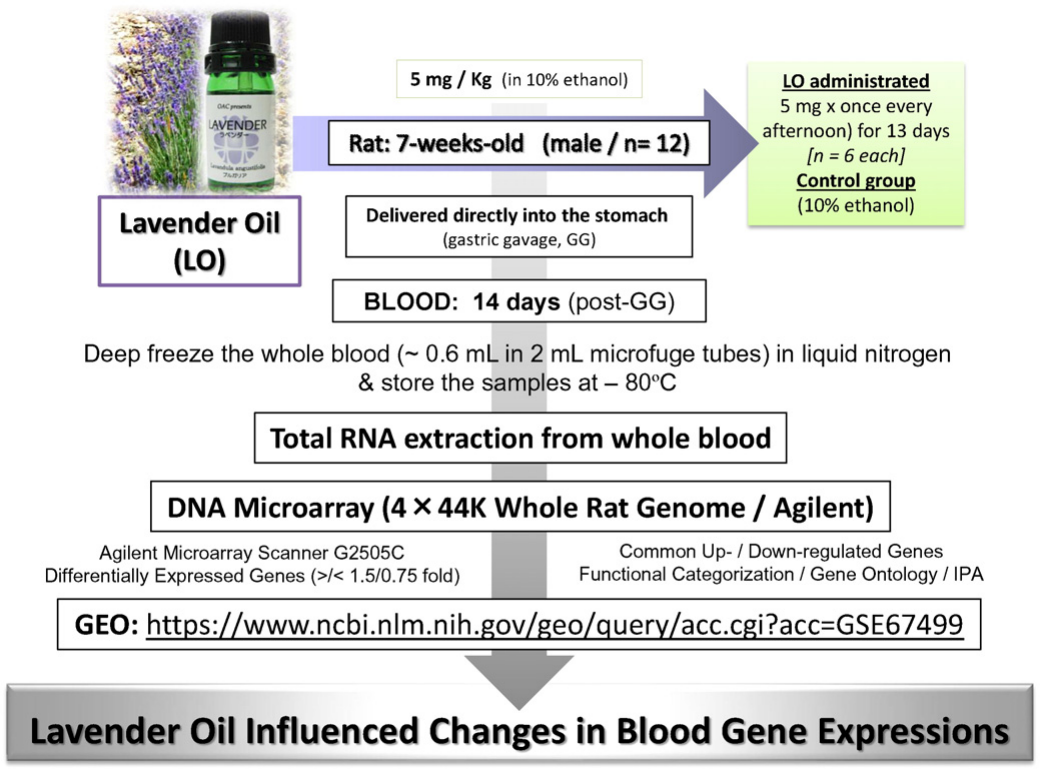
DNA microarray and bioinformatics analyses in the blood of rats after oral ingestion of the lavender oil (LO). Seven-week-old rats were orally administered the LO, once each day for 13 days, and on the 14th day, the blood from the heart was sampled. The blood whole-genome DNA microarray analysis was performed using the Agilent platform. Microarray data are publically available under the GEO series number GSE 67499. Differentially expressed genes were identified in the LO-treated blood sample followed by bioinformatics analyses as described in the text.

To continue our research forward into potential beneficial effects of LO, and to study the whole blood transcriptome influenced by LO ingestion, we used the experimental design and strategy as shown in Fig. 1. Essentially, following the LO rat model set up [1] and using the established DNA microarray approach in our laboratories, we have successfully extracted total RNA from freshly taken whole blood (deep-frozen) for use in downstream gene expression profiling, genome-wide, a first such report.

### Rat model, oral administration of LO, extraction of whole blood and total RNA isolation

Briefly, rats (Fischer, F334) were purchased from Japan SCL (Hamamatsu, Japan) and a total of 12 male rats (7 weeks old, ~ 142.8 to 167.5-g body weight) were housed at the Animal Institution in Showa University. Individual cages were used to maintain the rats in isolated animal rooms with controlled temperature and relative humidity with a 12 h light/12 h dark schedule (lights turned on at 08:00 AM). Rats had access to laboratory chow and tap water ad libitum. At least one-week acclimation period was allowed for the rats in the animal room prior to the conduct of the experiment. All of the animal care and experimental procedures were approved by the Institutional Animal Care and Use Committee of Showa University (approval number M6031).

The essential oil of lavender (LO, *L. angustifolia*, Biofloral, France) was administered to rats (n = 6 each group: control and LO treatment) directly into the stomach at a dose of 0 (10% ethanol; diluent: control group) or 5 mg/kg (in 10% ethanol: LO-administered group) once a day in the afternoon (11:25–13:50) for 13 days. Fourteen days after treatment, the rats were dissected under ether anesthesia and the whole blood was taken directly from the heart. The whole blood was put into 2 mL sterile Eppendorf tubes, which were then quickly immersed in liquid nitrogen (N_2_) before being stored in a − 80 °C deep freezer for downstream analysis. To note, the whole blood indicates no centrifugation to separate into plasma and serum prior to storage.

**Fig. 2:**
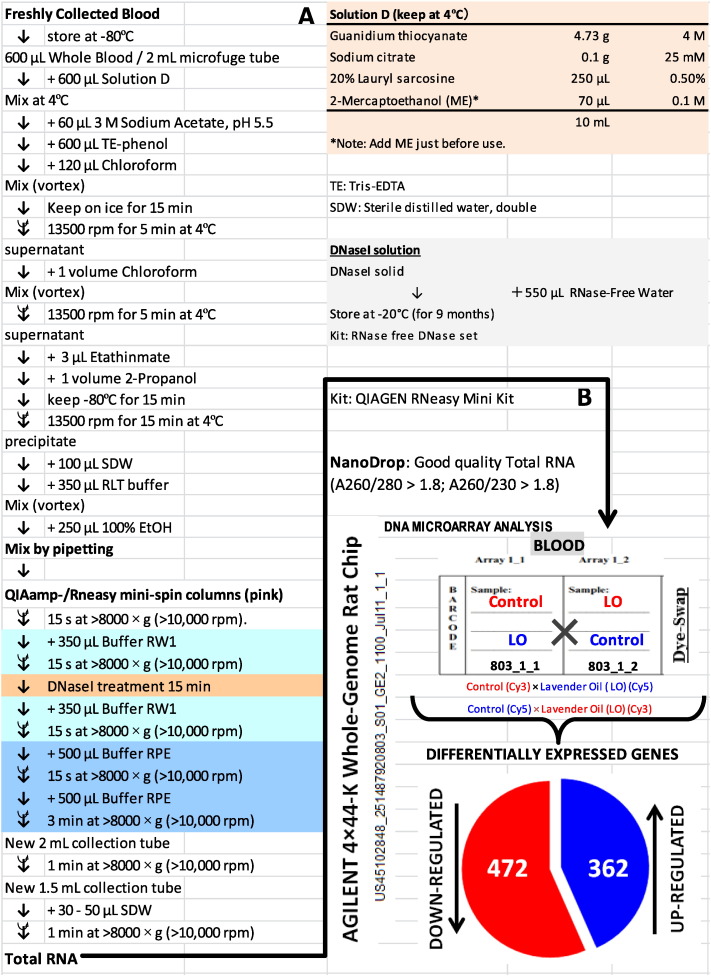
Rat whole blood total RNA extraction protocol, and DNA microarray analysis after oral ingestion of the lavender oil (LO) reveals 362 up-regulated and 472 down-regulated genes. (A) Blood was sampled as described in Fig. 1, and an optimized guanidium thiocynate method was used for total RNA extraction from deep frozen whole blood. (B) A two-color dye swap approach was employed for confident gene expression profiling in the blood of LO treated rat using the Agilent 4 × 44 K DNA microarray chip.

The microfuge tube containing the deep-frozen whole blood was placed on the bench at room temperature, and around 0.6 mL of the blood was removed to a new sterile microfuge tube, and immediately processed for total RNA extraction as schematically presented in detail in Fig. 2A. Briefly, the guanidium thiocynate method was used in combination with the QIAamp spin columns (QIAGEN RNeasy Mini Kit, QIAGEN, Maryland, MD, USA), which have been used downstream of the first individual RNA extraction step depending on each tissue [1], [3], [4], [5], [6], [7].

### 2.3 Whole blood whole-genome DNA microarray analysis

The extracted whole blood total RNA for each control and LO-administered sample was pooled [1], [15], [16] in each group prior to DNA microarray analysis, which was performed using a Rat whole genome 4 × 44-K DNA chip (G4131F; Agilent Technologies, Palo Alto, CA, USA) (Fig. 2B). Briefly, the total RNA (200 ng) was labeled with either Cy3 or Cy5 dye using an Agilent Low-RNA-Input Fluorescent Linear Amplification Kit (Agilent Technologies). The fluorescently labeled targets of the control as well as the LO-treated samples were hybridized to the same microarray slide with 60-mer probes. A flip-labeling (dye-swap or reverse labeling with Cy3 and Cy5 dyes) procedure was followed to nullify the dye bias that was associated with the unequal incorporation of the two Cy dyes into cDNA. The use of a dye-swap approach provides more-stringent selection conditions for changed gene expression profiling than the use of a simple single/two-color approach [6]. For the selection of differentially expressed genes by the dye-swap approach, we considered genes that were up-regulated in chip 1 (Cy3/Cy5 label for control and LO-administered samples, respectively) but down-regulated in chip 2 (Cy3/Cy5 label for LO-administered and control samples, respectively).

Hybridization and wash processes were performed according to the manufacturer's instructions, and the hybridized microarrays were scanned using an Agilent Microarray scanner G2505C. To detect significantly differentially expressed genes between the control and treated samples, each slide image was processed using Agilent Feature Extraction software (version 11.0.1.1). This program measures the Cy3 and Cy5 signal intensities of whole probes. Dye bias tends to be signal-intensity-dependent; therefore, the software selected probes using a set-by-rank consistency filter for dye normalization. Said normalization was performed by LOWESS (locally weighted linear regression), which calculates the log ratio of dye-normalized Cy3 and Cy5 signals and the final error of the log ratio. Statistical significance of the log ratio values obtained for individual microarray probes was calculated using the “most conservative error model” default function of the Feature Extraction software, by which both a propagated and a universal error model are evaluated, and the higher (more conservative estimate of error) p-value of two error models was reported [6; Agilent Technologies]. For the analyses, the threshold of significant differentially expressed genes was < 0.01 (for the confidence that the feature was not differentially expressed). Additionally, the erroneous data generated due to artifacts were eliminated before data analysis by the software.

### 2.4 Functional categorization of differentially expressed genes in the whole blood after LO ingestion and ingenuity pathway analysis of the whole blood transcriptome

We performed the pathway and disease state-focused gene classification of the differentially expressed whole blood genes based on the available categories of more than 100 biological pathways or specific disease states in the SABiosciences PCR array list (QIAGEN; www.sabiosciences.com) for *Rattus norvegicus*.

Biological function and network analyses were performed using Ingenuity Pathway Analysis (IPA; Ingenuity® Systems, www.ingenuity.com) tool. The dataset from the microarray (LO treatment in the whole blood), which includes the differentially expressed (≧/≦ 1.5/0.75-fold compared with control) genes and their corresponding fold change values, was uploaded as an Excel spread sheet into the IPA tool. To create gene networks, the genes were overlaid onto a global molecular network that was developed from information that was contained in the ingenuity knowledge base. The functional analysis identified the biological functions that were most significant to the dataset (p-value < 0.05) according to a right-tailed Fisher's exact test.

## 3 Discussion

### 3.1 Differentially expressed genes in the whole blood of rat after LO treatment

Utilizing an optimized total RNA extraction protocol for the whole blood, from rat orally administered LO, and as described in Fig. 2A, the two-color dye-swap DNA microarray analysis revealed 834 differentially expressed genes in the whole blood of rat given LO. Whole blood is an important target for analysis, as the sample can be immediately frozen or extracted without separation into plasma-serum, and the gene expressions unraveled in the blood can be compared with other tissues/organs, such as liver, spleen, and brain those of which are also our interest. As also mentioned above, extraction of good quality total RNA from the blood is not easy, especially as a critical requirement in high-throughput omics-based microarray approaches, a protocol for total RNA extraction from whole deep-frozen blood was optimized and used in this present study (Fig. 2A). Among these, 362 genes were up-regulated and 472 genes were down-regulated (Fig. 2B). These genes are listed in Supplementary Table 1. The outputs (raw data) of the microarray analysis that were used in this study are open and freely available under the number series GSE 67499 (whole genome) at the NCBI Gene Expression Omnibus (GEO) public functional genomics data repository.

### 3.2 Bioinformatics analysis provides a hint on the function and networks of genes in LO treated rat whole blood

**Fig. 3:**
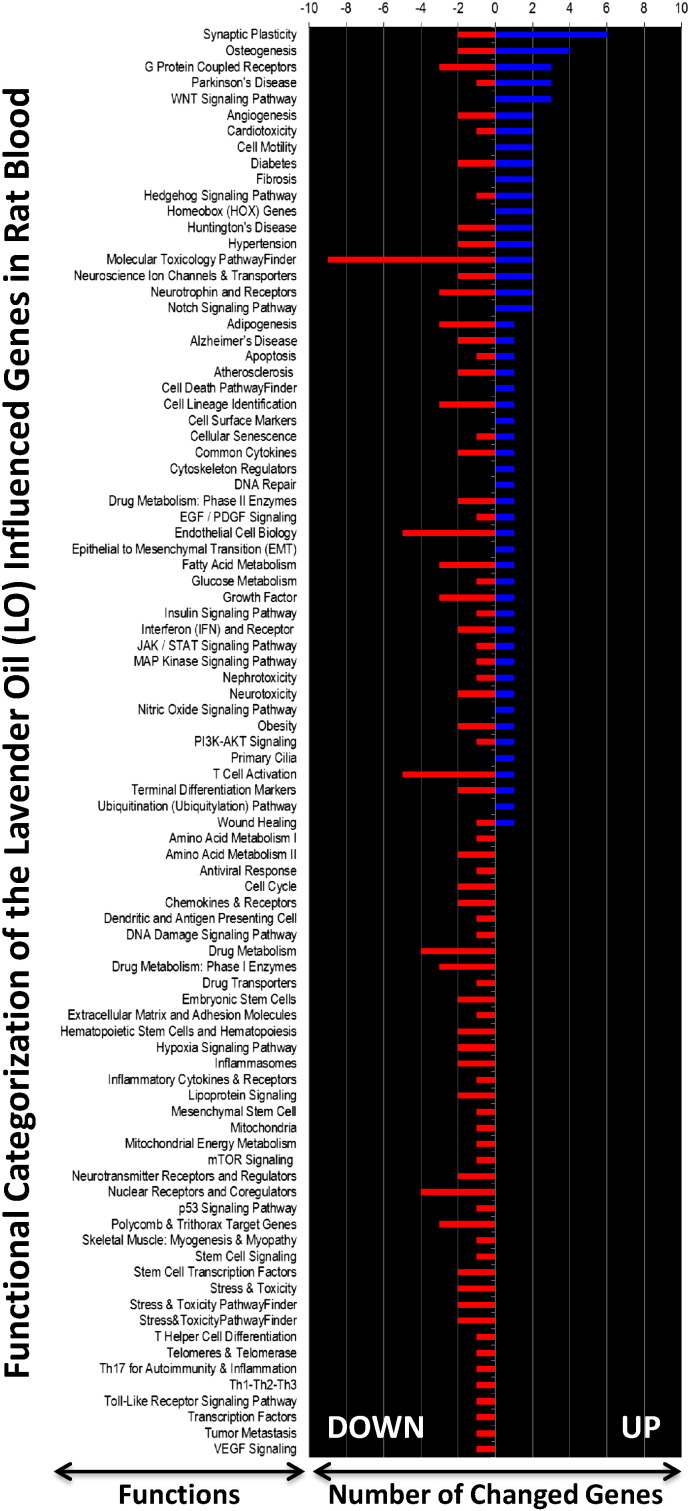
Pathway- and disease states-focused gene classification of genes in the whole blood of LO-treated rats. The genes (up-regulated — blue; down-regulated — red) were classified based on the available categories of more than 100 biological pathways or specific disease states in the SABiosciences PCR array list (QIAGEN; www.sabiosciences.com) for *Rattus norvegicus*. The gene functions are indicated on the left-hand side, and the numbers on the top of the chart represent the number of genes in each functional category.

**Fig. 4:**
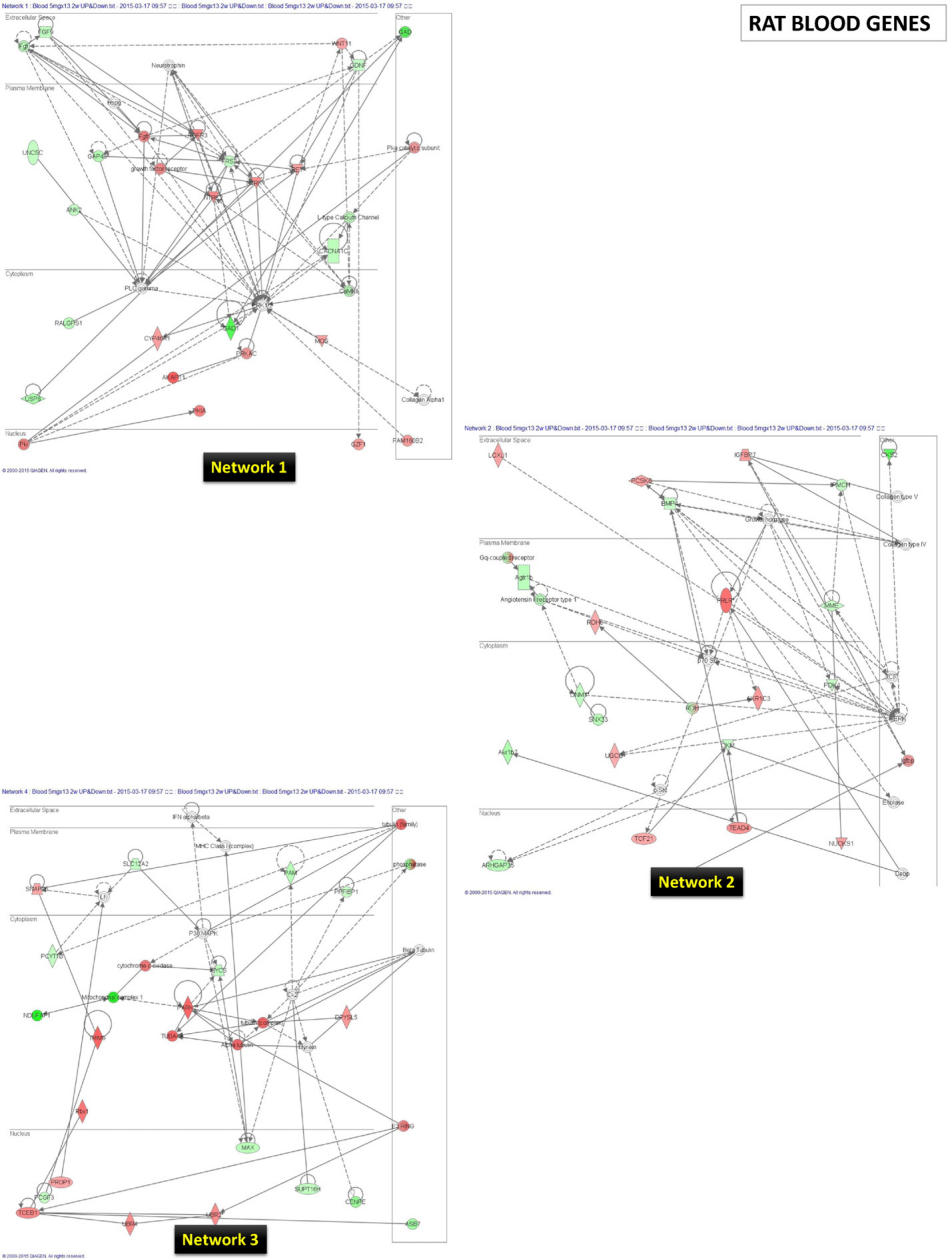
The top three networks (1, 2, and 3) for the rat whole blood under LO treatment by IPA analysis. The genes (up-regulated — red; down-regulated — green) are networked based on the evidence in the IPA.

**Fig. 5:**
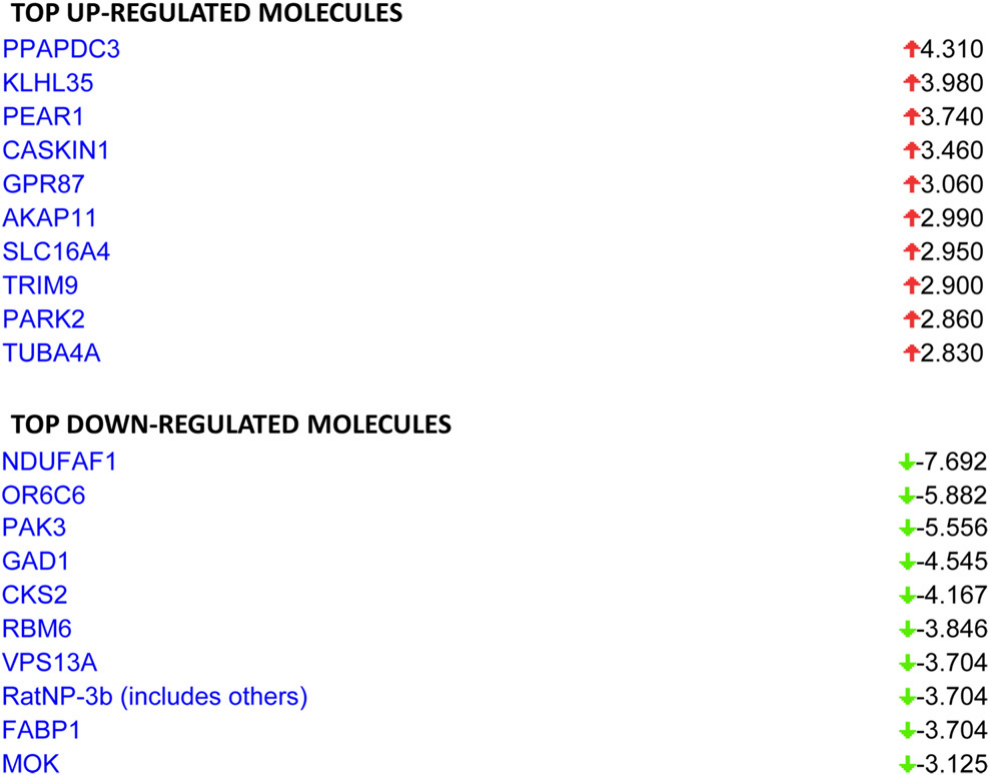
The top molecules for the rat whole blood under LO treatment by IPA analysis. The genes (up-regulated — red; down-regulated — green) are based on the evidence in the IPA.

The SABiosciences PCR array list (QIAGEN) was utilized to functionally categorize the differentially expressed whole blood genes, the results of which are presented graphically in Fig. 3. The annotated gene function and the genes description contained within each functional category are shown for the up-regulated (Supplementary Table 2) and down-regulated (Supplementary Table 3) genes. The number of down regulated genes was more highly annotated than the up-regulated genes, where some functions were more represented than others. For example, in the up-regulated gene list, synaptic plasticity, osteogenesis and hypertension were the categories with the most annotated genes. In the case of down-regulated gene list, molecular toxicology pathway finder, endothelial cell biology and nuclear receptors and coregulators were the categories with the most annotated genes. For a wider bioinformatics analysis, the biological function and network analysis was performed using the IPA tool. Numerous networks and pathways that were generated by this analysis revealed that LO can affect the blood in complex ways. For example, the genes influenced by LO in the top three networks (1, 2, and 3) are presented in Fig. 4. The blood IPA analysis also provided a list of top molecules that are gene candidates for the potential mechanism/s underlying the LO effects in the rat model (Fig. 5).

The top-regulated gene was *Ppapdc3*, also known as phospholipid phosphatase 7 (*plpp7*). Although, there is no research indicating its expression under essential oils, a literature review showed that it is a nuclear envelope protein which is involved in the signaling process, functioning as a negative regulator of myoblast differentiation [17]. The *Ndufaf1* (NADH dehydrogenase (ubiquinone) 1α-subcomplex, assembly factor 1) gene was the most down-regulated factor. *Ndufaf1* encodes a complex I assembly factor protein. Although it is an important protein for mitochondrial function, we do not know the reason for its decrease in the blood after LO treatment.

Lavender oil (LO) is used as a traditional medicine in aromatherapy for a wide variety of stresses. However, the molecular basis for its beneficial effects remains unclear. This study provides researchers with a new resource in terms of genes that are up- or down-regulated in the whole blood. As identification of these genes are a first such report from the blood of rat, our results also confirm the importance of a DNA microarray analysis approach in shedding light behind the use of LO and its effects on the body. Numerous gene candidates identified here will form the basis for future research and to compare with the genes in other tissues/organs of the rat, for a proper understanding of their function under LO.

The following are the supplementary data related to this article.

Supplementary Table 1 The up- and down-regulated whole blood genes following oral ingestion of lavender oil (LO). Supplementary Table 1

Supplementary Table 2 Functional categorization of the up-regulated genes in the rat whole blood post lavender oil treatment. Supplementary Table 2

Supplementary Table 3 Functional categorization of the down-regulated genes in the rat whole blood post lavender oil treatment. Supplementary Table 3

## Conflict of interest

The authors declare no conflict of interests.
